# Poly[[chloridodimethanol(μ_3_-pyridine-2,3-dicarboxyl­ato)europium(III)] methanol monosolvate]

**DOI:** 10.1107/S1600536812017862

**Published:** 2012-04-28

**Authors:** Dayu Wu, Liyang Chen, Genhua Wu

**Affiliations:** a School of Chemistry and Chemical Engineering, Anqing Teachers College, Anqing 246011, People’s Republic of China

## Abstract

The asymmetric unit of the title compound, {[Eu(C_7_H_3_NO_4_)Cl(CH_3_OH)_2_]·CH_3_OH}_*n*_, contains one Eu^III^ ion, one pyridine 2,3-dicarboxylate dianion (PDC), two CH_3_OH mol­ecules coordinating to the metal atom, one coordinating chloride and one lattice occluded CH_3_OH mol­ecule. In the crystal, each PDC anion coordinates to three adjacent Eu^III^ ions by the pyridine N and O atoms of the carboxyl­ate groups. The Eu^III^ cation is eightfold coordinated by four carboxyl­ate O atoms, one pyridine N atom, two MeOH and one chloride anion in the form of a distorted polyhedron. Extended coordination of the PDC ligand lead to the formation of a two-dimensional coordination polymer parallel to (10-1).

## Related literature

For related work on pyridine-carboxyl­ate transition-metal compounds, see: Swamy *et al.* (1998 ▶); Zhong *et al.* (1994 ▶); Zhang *et al.* (2003 ▶); Wu *et al.* (2003 ▶); Tong *et al.* (2000 ▶). For work on lanthanide compounds, see, for example: Zhao *et al.* (2004 ▶). 
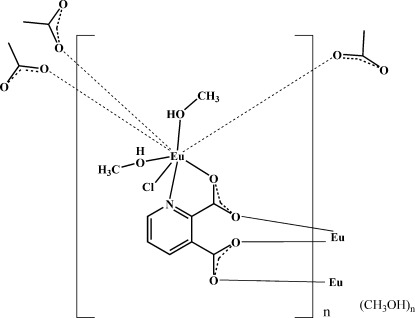


## Experimental

### 

#### Crystal data

[Eu(C_7_H_3_NO_4_)Cl(CH_4_O)_2_]·CH_4_O
*M* *_r_* = 448.64
Monoclinic, 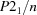

*a* = 10.4870 (3) Å
*b* = 10.9123 (3) Å
*c* = 12.9248 (3) Å
β = 99.694 (2)°
*V* = 1457.96 (7) Å^3^
*Z* = 4
Mo *K*α radiation
μ = 4.51 mm^−1^
*T* = 150 K
0.20 × 0.15 × 0.14 mm

#### Data collection

Bruker SMART CCD area-detector diffractometer
Absorption correction: multi-scan (*SADABS*; Bruker, 1997 ▶) *T*_min_ = 0.448, *T*_max_ = 0.532
7658 measured reflections
2550 independent reflections
2260 reflections with *I* > 2σ(*I*)
*R*_int_ = 0.029

#### Refinement

*R*[*F* ^2^ > 2σ(*F* ^2^)] = 0.026
*wR*(*F* ^2^) = 0.057
*S* = 1.02
2550 reflections
184 parameters
H-atom parameters constrained
Δρ_max_ = 1.34 e Å^−3^
Δρ_min_ = −0.56 e Å^−3^

### 

Data collection: *SMART* (Bruker, 1997 ▶); cell refinement: *SAINT* (Bruker, 1997 ▶); data reduction: *SAINT*; program(s) used to solve structure: *SHELXS97* (Sheldrick, 2008 ▶); program(s) used to refine structure: *SHELXL97* (Sheldrick, 2008 ▶); molecular graphics: *SHELXTL* (Sheldrick, 2008 ▶); software used to prepare material for publication: *SHELXL97* and *PLATON* (Spek, 2009 ▶).

## Supplementary Material

Crystal structure: contains datablock(s) I, global. DOI: 10.1107/S1600536812017862/ds2185sup1.cif

Structure factors: contains datablock(s) I. DOI: 10.1107/S1600536812017862/ds2185Isup2.hkl

Additional supplementary materials:  crystallographic information; 3D view; checkCIF report

## Figures and Tables

**Table 1:** Selected bond lengths (Å)

Eu1—O3^i^	2.344 (3)
Eu1—O4	2.346 (3)
Eu1—O2	2.354 (3)
Eu1—O1^ii^	2.373 (3)
Eu1—O5	2.450 (3)
Eu1—O6	2.490 (3)
Eu1—N1^i^	2.655 (3)
Eu1—Cl1	2.7723 (11)

Symmetry codes: (i) 
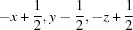
; (ii) 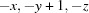.

**Fig. 1.**
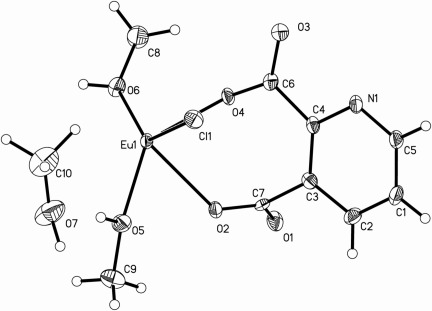
The molecular structure of the title compound, the thermal ellipsoids were drawn at 50% probability level.

**Fig. 2.**
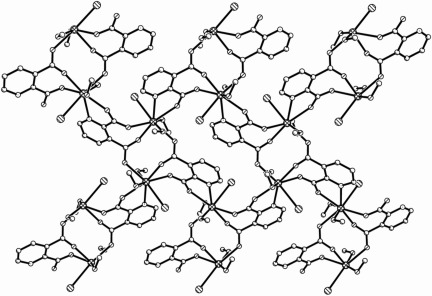
The 2-D packing structure of the title compound.

## supplementary crystallographic information

### Comment

The pyridine dicarboxylic acid (H_2_pydc) is a diverse bridging ligand and has drawn some attention in photochemistry and crystal engineering. In case of pyridine-2,3-dicarboxylate functional groups, pydc exhibits bis(monodentate) (Swamy *et al.*,1998), tridentate(Zhong *et al.*, 1994; Zhang *et al.*, 2003), bis(bidentate) (Wu *et al.*, 2003; Tong *et al.*, 2000) coordination modes to form a few transition metal coordination polymers. It should display high reactivity to the lanthanide, since the oxygen atom of carboxylate group has a strong affinity to Ln^3+^ ions (Zhao *et al.*,2004). In this work, we selected pyridine-2,3-dicarboxylate and lanthanide ions to assemble coordination polymer under hydrothermal conditions, forming two-dimensional network structures. As depicted in Figure 1, the asymmetric unit contains one europium ion, one deprotonated pyridine 2,3-dicarboxylate ligand (PDC), two CH_3_OH molecules coordinated to the metal center, one coordinated chloride and one lattice occluded CH_3_OH molecule. In the crystal structure, each PDC ligand coordinates to three adjacent Eu ions by the pyridyl N and O atoms of the carboxylate moieties. Eu is coordinated by four O atoms of carboxylate, one pyridyl N, two MeOH and one chloride dispaying eight-coordinated environment with average Eu—O bond length of being 2.3825 Å, obviously shorter than that of Eu—Cl 2.7724 Å (Table 1). Extended coordination of PDC ligand lead to the formation of a 2D coordination polymer (Figure 2). π···π stacking interactions were found to stabilize two-dimensional structure with the shortest carbon···carbon distance of 3.487 Å in the plan.

### Experimental

A mixture of EuCl_3_^.^6H_2_O (0.037 g, 0.1 mmol), H_2_pydc (0.017 g, 0.1 mmol) in 8 mL H_2_O was stirred at room temperature for 30 min, the solution was put into a 25 mL Teflon-lined stainless-steel container, heated to 165 ^o^C and maintained for 48 h, and then cooled to room temperature in 45 h. The colorless block crystal was obtained by filtration, washed with water and ethanol in 67% yield (based on Eu). Anal. Calc. for C_10_ H_15_ Cl Eu N O_7_ : C, 26.77; H, 3.37; N, 3.12. Found: C, 27.05; H, 3.47; N, 3.16%. IR (KBr pellet): 3407(m), 1698(m), 1594(s), 1565(s), 1442(m), 1381(s), 1254(m), 1095(m), 758(m) cm^-1^.

### Refinement

C-bound H atoms were placed geometrically and allowed to ride during refinement with C—H = 0.93–0.96 Å with *U*_iso_(H) = 1.2 *U*_eq_(C). The hydroxy H atom of the methanol solvent molecule was located in a difference Fourier map and refined as riding with the parent atom with *U*_iso_(H) = 1.5*U*_eq_(O), O—H distances 0.82 and 0.85 Å.

### Crystal data

**Table:**

[Eu(C_7_H_3_NO_4_)Cl(CH_4_O)_2_]·CH_4_O	*F*(000) = 872
*M**_r_* = 448.64	*D*_x_ = 2.044 Mg m^−^^3^
Monoclinic, *P*2_1_/*n*	Mo *K*α radiation, λ = 0.71073 Å
Hall symbol: -P 2yn	Cell parameters from 5415 reflections
*a* = 10.4870 (3) Å	θ = 3.0–29.1°
*b* = 10.9123 (3) Å	µ = 4.51 mm^−^^1^
*c* = 12.9248 (3) Å	*T* = 150 K
β = 99.694 (2)°	Block, white
*V* = 1457.96 (7) Å^3^	0.20 × 0.15 × 0.14 mm
*Z* = 4	

### Data collection

**Table:**

Bruker SMART CCD area-detector diffractometer	2550 independent reflections
Radiation source: fine-focus sealed tube	2260 reflections with *I* > 2σ(*I*)
Graphite monochromator	*R*_int_ = 0.029
phi and ω scans	θ_max_ = 25.0°, θ_min_ = 3.0°
Absorption correction: multi-scan (*SADABS*; Bruker, 1997)	*h* = −12→11
*T*_min_ = 0.448, *T*_max_ = 0.532	*k* = −12→12
7658 measured reflections	*l* = −14→15

### Refinement

**Table:**

Refinement on *F*^2^	Primary atom site location: structure-invariant direct methods
Least-squares matrix: full	Secondary atom site location: difference Fourier map
*R*[*F*^2^ > 2σ(*F*^2^)] = 0.026	Hydrogen site location: inferred from neighbouring sites
*wR*(*F*^2^) = 0.057	H-atom parameters constrained
*S* = 1.02	*w* = 1/[σ^2^(*F*_o_^2^) + (0.0287*P*)^2^ + 1.5721*P*] where *P* = (*F*_o_^2^ + 2*F*_c_^2^)/3
2550 reflections	(Δ/σ)_max_ = 0.001
184 parameters	Δρ_max_ = 1.34 e Å^−^^3^
0 restraints	Δρ_min_ = −0.56 e Å^−^^3^

### Special details

**Table:**

Geometry. All esds (except the esd in the dihedral angle between two l.s. planes) are estimated using the full covariance matrix. The cell esds are taken into account individually in the estimation of esds in distances, angles and torsion angles; correlations between esds in cell parameters are only used when they are defined by crystal symmetry. An approximate (isotropic) treatment of cell esds is used for estimating esds involving l.s. planes.
Refinement. Refinement of F^2^ against ALL reflections. The weighted R-factor wR and goodness of fit S are based on F^2^, conventional R-factors R are based on F, with F set to zero for negative F^2^. The threshold expression of F^2^ > 2sigma(F^2^) is used only for calculating R-factors(gt) etc. and is not relevant to the choice of reflections for refinement. R-factors based on F^2^ are statistically about twice as large as those based on F, and R- factors based on ALL data will be even larger.

### Fractional atomic coordinates and isotropic or equivalent isotropic displacement parameters (Å^2^)

**Table:**

	*x*	*y*	*z*	*U*_iso_*/*U*_eq_	
Eu1	0.19538 (2)	0.471828 (18)	0.142087 (15)	0.01375 (8)	
C1	0.1686 (5)	1.0083 (4)	−0.0245 (4)	0.0217 (10)	
H1A	0.1752	1.0679	−0.0747	0.026*	
C2	0.1143 (4)	0.8967 (4)	−0.0540 (3)	0.0204 (10)	
H2A	0.0830	0.8801	−0.1243	0.024*	
C3	0.1066 (4)	0.8081 (4)	0.0227 (3)	0.0152 (9)	
C4	0.1520 (4)	0.8388 (4)	0.1267 (3)	0.0141 (9)	
C5	0.2135 (4)	1.0315 (4)	0.0803 (3)	0.0187 (9)	
H5A	0.2508	1.1073	0.0992	0.022*	
C6	0.1440 (4)	0.7542 (4)	0.2180 (3)	0.0142 (9)	
C7	0.0458 (4)	0.6855 (4)	−0.0113 (3)	0.0149 (9)	
C8	−0.0245 (5)	0.4395 (4)	0.3294 (4)	0.0309 (12)	
H8A	−0.1158	0.4238	0.3164	0.046*	
H8B	−0.0096	0.5262	0.3342	0.046*	
H8C	0.0137	0.4012	0.3942	0.046*	
C9	0.2592 (5)	0.3714 (5)	−0.1019 (4)	0.0298 (12)	
H9A	0.3221	0.3790	−0.1476	0.045*	
H9B	0.1840	0.4189	−0.1292	0.045*	
H9C	0.2353	0.2869	−0.0974	0.045*	
C10	0.6250 (6)	0.3148 (5)	0.1466 (4)	0.0437 (14)	
H10A	0.7143	0.3136	0.1383	0.066*	
H10B	0.6088	0.2489	0.1919	0.066*	
H10C	0.6057	0.3915	0.1769	0.066*	
Cl1	0.41214 (10)	0.62123 (9)	0.17815 (8)	0.0207 (2)	
N1	0.2053 (3)	0.9493 (3)	0.1559 (3)	0.0161 (8)	
O1	−0.0723 (3)	0.6882 (2)	−0.0493 (2)	0.0201 (7)	
O2	0.1171 (3)	0.5928 (2)	−0.0058 (2)	0.0189 (7)	
O3	0.1826 (3)	0.7950 (2)	0.3084 (2)	0.0180 (7)	
O4	0.0993 (3)	0.6474 (2)	0.2001 (2)	0.0154 (6)	
O5	0.3136 (3)	0.4156 (3)	0.0010 (2)	0.0222 (7)	
O6	0.0332 (3)	0.3900 (3)	0.2445 (2)	0.0235 (7)	
O7	0.5459 (4)	0.3005 (4)	0.0477 (3)	0.0470 (10)	
H7A	0.5784	0.3203	−0.0159	0.056*	
H5B	0.3905	0.3848	0.0202	0.056*	
H6B	0.0268	0.3152	0.2582	0.056*	

### Atomic displacement parameters (Å^2^)

**Table:**

	*U*^11^	*U*^22^	*U*^33^	*U*^12^	*U*^13^	*U*^23^
Eu1	0.01652 (13)	0.00914 (13)	0.01382 (12)	−0.00020 (9)	−0.00258 (8)	0.00020 (8)
C1	0.029 (3)	0.013 (2)	0.021 (2)	0.0009 (19)	0.001 (2)	0.0053 (18)
C2	0.024 (3)	0.019 (2)	0.015 (2)	−0.0010 (19)	−0.0036 (18)	0.0026 (18)
C3	0.012 (2)	0.016 (2)	0.017 (2)	0.0029 (18)	−0.0012 (16)	−0.0021 (17)
C4	0.011 (2)	0.011 (2)	0.018 (2)	0.0013 (17)	−0.0040 (17)	−0.0012 (17)
C5	0.024 (3)	0.011 (2)	0.019 (2)	−0.0006 (19)	−0.0025 (18)	−0.0003 (18)
C6	0.008 (2)	0.015 (2)	0.018 (2)	0.0021 (17)	−0.0011 (17)	0.0019 (17)
C7	0.021 (3)	0.015 (2)	0.007 (2)	0.0018 (19)	−0.0006 (17)	0.0021 (16)
C8	0.036 (3)	0.029 (3)	0.031 (3)	0.005 (2)	0.017 (2)	0.003 (2)
C9	0.031 (3)	0.033 (3)	0.024 (3)	−0.004 (2)	0.002 (2)	−0.008 (2)
C10	0.030 (3)	0.052 (4)	0.050 (4)	0.001 (3)	0.006 (3)	0.012 (3)
Cl1	0.0198 (6)	0.0199 (5)	0.0212 (6)	−0.0041 (4)	0.0000 (4)	−0.0028 (4)
N1	0.017 (2)	0.0103 (18)	0.0194 (19)	0.0012 (14)	−0.0013 (15)	−0.0002 (14)
O1	0.0193 (18)	0.0143 (15)	0.0243 (17)	−0.0021 (13)	−0.0027 (13)	−0.0026 (12)
O2	0.0263 (18)	0.0117 (15)	0.0163 (15)	0.0037 (14)	−0.0034 (13)	−0.0013 (12)
O3	0.0208 (18)	0.0147 (15)	0.0166 (16)	−0.0028 (13)	−0.0024 (13)	−0.0004 (12)
O4	0.0181 (17)	0.0090 (14)	0.0177 (15)	−0.0007 (12)	−0.0006 (12)	−0.0019 (12)
O5	0.0222 (18)	0.0244 (17)	0.0182 (16)	0.0044 (14)	−0.0019 (13)	−0.0003 (13)
O6	0.0294 (19)	0.0183 (16)	0.0224 (17)	−0.0005 (14)	0.0032 (14)	0.0033 (13)
O7	0.043 (3)	0.067 (3)	0.034 (2)	0.018 (2)	0.0145 (19)	0.0166 (19)

### Geometric parameters (Å, º)

**Table:**

Eu1—O3^i^	2.344 (3)	C7—O1	1.253 (5)
Eu1—O4	2.346 (3)	C7—O2	1.253 (5)
Eu1—O2	2.354 (3)	C8—O6	1.445 (5)
Eu1—O1^ii^	2.373 (3)	C8—H8A	0.9600
Eu1—O5	2.450 (3)	C8—H8B	0.9600
Eu1—O6	2.490 (3)	C8—H8C	0.9600
Eu1—N1^i^	2.655 (3)	C9—O5	1.438 (5)
Eu1—Cl1	2.7723 (11)	C9—H9A	0.9600
C1—C2	1.371 (6)	C9—H9B	0.9600
C1—C5	1.380 (6)	C9—H9C	0.9600
C1—H1A	0.9300	C10—O7	1.410 (7)
C2—C3	1.397 (6)	C10—H10A	0.9600
C2—H2A	0.9300	C10—H10B	0.9600
C3—C4	1.389 (6)	C10—H10C	0.9600
C3—C7	1.515 (6)	N1—Eu1^iii^	2.655 (3)
C4—N1	1.355 (5)	O1—Eu1^ii^	2.373 (3)
C4—C6	1.512 (6)	O3—Eu1^iii^	2.344 (3)
C5—N1	1.340 (5)	O5—H5B	0.8701
C5—H5A	0.9300	O6—H6B	0.8404
C6—O3	1.253 (5)	O7—H7A	0.9656
C6—O4	1.262 (5)		
			
O3^i^—Eu1—O4	146.02 (10)	N1—C5—H5A	118.7
O3^i^—Eu1—O2	141.52 (10)	C1—C5—H5A	118.7
O4—Eu1—O2	72.05 (9)	O3—C6—O4	123.4 (4)
O3^i^—Eu1—O1^ii^	75.98 (10)	O3—C6—C4	117.3 (3)
O4—Eu1—O1^ii^	122.41 (10)	O4—C6—C4	119.2 (3)
O2—Eu1—O1^ii^	85.21 (10)	O1—C7—O2	125.6 (4)
O3^i^—Eu1—O5	71.06 (10)	O1—C7—C3	115.6 (4)
O4—Eu1—O5	137.83 (10)	O2—C7—C3	118.6 (4)
O2—Eu1—O5	71.76 (10)	O6—C8—H8A	109.5
O1^ii^—Eu1—O5	74.93 (10)	O6—C8—H8B	109.5
O3^i^—Eu1—O6	87.13 (10)	H8A—C8—H8B	109.5
O4—Eu1—O6	75.79 (10)	O6—C8—H8C	109.5
O2—Eu1—O6	117.19 (10)	H8A—C8—H8C	109.5
O1^ii^—Eu1—O6	69.05 (10)	H8B—C8—H8C	109.5
O5—Eu1—O6	141.48 (10)	O5—C9—H9A	109.5
O3^i^—Eu1—N1^i^	63.22 (10)	O5—C9—H9B	109.5
O4—Eu1—N1^i^	83.10 (10)	H9A—C9—H9B	109.5
O2—Eu1—N1^i^	151.18 (10)	O5—C9—H9C	109.5
O1^ii^—Eu1—N1^i^	121.25 (10)	H9A—C9—H9C	109.5
O5—Eu1—N1^i^	123.02 (10)	H9B—C9—H9C	109.5
O6—Eu1—N1^i^	68.52 (10)	O7—C10—H10A	109.5
O3^i^—Eu1—Cl1	92.51 (7)	O7—C10—H10B	109.5
O4—Eu1—Cl1	81.38 (7)	H10A—C10—H10B	109.5
O2—Eu1—Cl1	88.32 (8)	O7—C10—H10C	109.5
O1^ii^—Eu1—Cl1	151.46 (8)	H10A—C10—H10C	109.5
O5—Eu1—Cl1	76.64 (7)	H10B—C10—H10C	109.5
O6—Eu1—Cl1	137.37 (7)	C5—N1—C4	117.8 (4)
N1^i^—Eu1—Cl1	73.41 (8)	C5—N1—Eu1^iii^	126.3 (3)
C2—C1—C5	119.5 (4)	C4—N1—Eu1^iii^	115.6 (2)
C2—C1—H1A	120.2	C7—O1—Eu1^ii^	126.7 (3)
C5—C1—H1A	120.2	C7—O2—Eu1	128.4 (2)
C1—C2—C3	119.2 (4)	C6—O3—Eu1^iii^	128.6 (3)
C1—C2—H2A	120.4	C6—O4—Eu1	130.2 (3)
C3—C2—H2A	120.4	C9—O5—Eu1	126.9 (3)
C4—C3—C2	117.9 (4)	C9—O5—H5B	109.8
C4—C3—C7	123.5 (4)	Eu1—O5—H5B	116.4
C2—C3—C7	118.5 (4)	C8—O6—Eu1	132.9 (3)
N1—C4—C3	122.8 (4)	C8—O6—H6B	98.2
N1—C4—C6	113.6 (3)	Eu1—O6—H6B	123.2
C3—C4—C6	123.6 (4)	C10—O7—H7A	120.6
N1—C5—C1	122.6 (4)		

Symmetry codes: (i) −*x*+1/2, *y*−1/2, −*z*+1/2; (ii) −*x*, −*y*+1, −*z*; (iii) −*x*+1/2, *y*+1/2, −*z*+1/2.
